# Selection and Multiplexing of Reverse Transcription–Quantitative PCR Tests Targeting Relevant Honeybee Viral Pathogens

**DOI:** 10.3390/microorganisms12061105

**Published:** 2024-05-29

**Authors:** Franca Rossi, Ilaria Del Matto, Luciano Ricchiuti, Lucio Marino

**Affiliations:** Istituto Zooprofilattico Sperimentale dell’Abruzzo e del Molise “G. Caporale”, Campo Boario, 64100 Teramo, Italy; i.delmatto@izs.it (I.D.M.); l.ricchiuti@izs.it (L.R.); l.marino@izs.it (L.M.)

**Keywords:** honeybee, viral pathogens, reverse transcription–quantitative PCR (RT-qPCR) tests, TaqMan probes, duplex reactions

## Abstract

Verifying the inclusivity of molecular detection methods gives indications about the reliability of viral infection diagnosis because of the tendency of viral pathogens to undergo sequence variation. This study was aimed at selecting inclusive probes based on reverse transcription–quantitative PCR (RT-qPCR) assays for the diagnosis of the most widespread and detrimental viruses infecting honeybees, namely the acute bee paralysis virus (ABPV), the black queen cell virus (BQCV), the chronic paralysis bee virus (CBPV), the deformed wing virus variants A (DWVA) and B (DWVB), and the sacbrood virus (SBV). Therefore, previously described detection methods were re-evaluated in silico for their specificity and inclusivity. Based on this evaluation, selected methods were modified, or new ones were designed and tested in duplex RT-qPCR reactions. The limits of detection (LODs), effect of multiplexing on sensitivity and the viral RNA quantification potential in bees and hive debris were assessed. This study made available diagnostic assays able to detect an increased number of virus variants compared with previously described tests and two viral pathogens in a single PCR reaction.

## 1. Introduction

Honeybee health is being threatened worldwide by different detrimental factors that have led to annual mortality rates ranging between 9.6 and 26% for the domesticated honeybees *Apis mellifera* and *A. cerana*, with the extent of these varying with the geographical area [1]. Different viral pathogens contribute to honeybee mortality by causing acute infections or by acting as biotic stressors [2,3]. In particular, colony losses for *A. mellifera*, the most important commercial pollinator, occur mainly over winter and have been strongly associated with deformed wing virus (DWV) transmission by *Varroa destructor*, since this allows direct access for the virus into the bee hemolymph, causing infections very rapidly. DWV is a good indicator of bee colony decline because of its positive temporal correlation with honeybee colony losses and has been shown to act as a pathogen driving bee mortality [4]. In addition, DWV induces immune suppression in bees, facilitating the ectoparasitic trophic activity of the *V. destructor* mite [3].

Viruses affecting honeybees are able to replicate in different organs with broad or restricted tropism and impair organ function when they are present at very high titers, giving rise to infection symptoms. Asymptomatic infections remain undetected and may cause long-term damage to honeybee colonies [5].

The most-often detected viruses in honeybees are the DWV, in the two variants A (DWVA) and B (DWVB), the latter originally named *Varroa destructor* virus-1 (VDV-1) [2,6]; the chronic bee paralysis virus (CBPV); the black queen cell virus (BQCV); the sacbrood virus (SBV) and the acute bee paralysis virus (ABPV) [2,7,8,9,10,11,12,13,14,15,16]. ABPV is the most common of the viruses causing acute paralysis, i.e., ABPV, Israeli acute paralysis virus (IAPV) and Kashmir bee virus (KBV), in Europe and South America and has been detected globally, with the exception of Australia [13,17,18,19]. IAPV was suspected but not confirmed to be responsible for the colony collapse disorder (CCD), characterized by a sudden loss of up to 90% hives in apiaries without a clear precedent of disease. However, the involvement of the closely related ABPV and KBV cannot be excluded due to the possibility of misidentification [20].

Among these most common viruses, DWVB and CBPV have shown increasing prevalence in many geographical areas [9,21,22,23,24,25]. In particular, the relative representation of DWVB in thousands of RNAseq libraries for *A. mellifera*, *Bombus terrestris* and *V. destructor* increased after 2014 in the US and became dominant in Europe [24].

The viruses DWVA, DWVB, BQCV, CBPV, SBV and ABPV are positive-sense single-stranded (+ss) RNA viruses of the families Dicistroviridae and Iflaviridae, except for CBPV, which remains unassigned [14,20]. These threaten beekeeping worldwide and give rise to a multiplicity of symptoms [20]. In particular, DWV-infected bees show crumpled or aborted wings, shortened abdomens, paralysis, severely shortened life span in workers and drones and impaired learning and foraging behavior. SBV causes pupation failure, the formation of swollen larvae (“sacs”) filled with ecdysial fluid containing numerous viral particles, impaired foraging and a reduction in adult life span and metabolic activities. BQCV affects queen larvae that become initially yellowish with a sac-appearance and then dark brown, and it causes the death of infected pupae and the significant reduction in the adult bee life span. CBPV-infected bees appear to be trembling, have a bloated abdomen, be unable to fly and crawl on the ground. Alternatively, bees appear hairless, darker and shiny, which are attacked by the healthy bees. In both cases, the infected bees die within a few days. ABPV infection manifests as darkened and hairless trembling bees that are unable to fly and that crawl on the ground before their rapid death. ABPV, DWVA and DWVB are transmitted by *V. destructor* [2]. Moreover, it was reported that *V. destructor* mite presence was significantly associated with a higher prevalence of BQCV, CBPV and SBV [26].

McMahon et al. [4] reported that DWVB is more virulent than DWVA in adult worker bees exposed to field-derived DWVA and DWVB in the absence of *V. destructor* and causes a faster colony collapse. On the contrary, Norton et al. [27] found evidence that the DWVB variant caused a lower mortality in honeybee pupae than DWVA and that it accumulated to higher levels. This might explain the increasing DWVB prevalence over DWVA reported in different countries [21,22,23,24,25]. Moreover, Kevill et al. [23] reported that DWVA is transmitted by *V. destructor*, while DWVB is not. In addition, the latter caused less severe infections than DWVA and might be able to protect bee colonies from DWVA infection by superinfection exclusion [23]. In addition, DWVB appeared to be detrimental for *V. destructor* since mites with high DWVB loads had an average lifespan significantly shorter than those with high DWVA levels or with low levels of DWVA or DWVB. This could be a consequence of the different abilities of DWVB and DWVA to replicate in *V. destructor*, though there is no general agreement on this aspect [28,29,30].

Based on the reported differences in pathogenicity between the DWVA and DWVB variants, the use of molecular detection methods able to differentiate between the two variants is preferable in studies regarding virulence and effects on bee colony health. However, many of the DWV-targeted RT-qPCR tests reported either do not discriminate between the two variants or target only the DWVA variant [11,15,31,32,33].

The RT-qPCR-based methods available for the detection of ABPV, BQCV, CBPV, DWVA, DWVB and SBV are singleplex tests that use Sybr Green [25,26,29,31,32,34,35,36,37,38] or fluorescent-probe-based detection [33,39,40,41,42], but only two of these have been validated [6,40].

Most tests have already been applied in ecological studies and diagnostic screenings [4,9,25,43,44,45,46,47,48].

For the DWV virus, the analysis of published genome sequences suggested that PCR/qPCR detection of the virus can be unreliable since variant detection depends on the choice of appropriate oligonucleotide primers [24]. Therefore, this study aimed at investigating in silico the ability of the described RT-qPCR tests for the viruses ABPV, BQCV, CBPV, DWVA, DWVB and SBV to be specific and inclusive. The ones theoretically more reliable were selected to derive new methods with improved inclusivity. For some of the viral pathogens, new methods were designed.

## 2. Materials and Methods

### 2.1. Oligonucleotide Selection/Design

The primers and probe systems previously described for the detection of honeybee viral pathogens ABPV, BQCV, DWVA, DWVB, CPBV and SBV [25,26,29,31,32,33,34,35,36,37,38,39] were tested for their specificity and inclusivity by BLASTN (https://blast.ncbi.nlm.nih.gov/Blast.cgi, accessed on 27 April 2024) against 500 database entries. Those matching the highest number of sequences of the target virus were selected for use in one-step RT-qPCR tests with TaqMan probes. When opportune, the primers were modified or newly designed to increase the number of matched sequences and to improve inclusivity. TaqMan probes were designed for tests originally conceived for Sybr Green detection. The number of sequences aligned for oligo design was 43 for ABPV, 59 for BQCV, 52 for CBPV, 110 for DWVA, 46 for DWVB and 106 for SBV. Only complete sequences of the target genes were considered for primer design, and all the included sequences had different BLASTN scores in the alignment, with a complete gene sequence used as the query. Primers and probes used in this study were synthetized by Eurofins Genomics (Ebersberg, Germany). Their tendency to dimerize and their optimal use in qPCR tests were analyzed by the online tools available from Eurofins Genomics (https://eurofinsgenomics.eu/en/dna-rna-oligonucleotides/oligo-tools, accessed on 23 December 2023).

### 2.2. RNA Extraction

RNA was extracted from spiked aliquots of two honeybees or from approximately 800 µL of hive debris transferred into 2 mL Eppendorf safe-lock tubes (Eppendorf, Milan, Italy) containing approximately 200 µL of unwashed glass beads of 200 µm diameter (Merck, Darmstadt, Germany) that had been sterilized by autoclaving. In these tubes, the hive debris was added until it reached the 1 mL graduation mark of the tube. Weighing of the hive debris was not carried out since its practical significance is limited due to the extremely high variability in composition and density.

One mL of Macherey Nagel Nucleozol reagent (Carlo Erba, Cornaredo, Italy) was added. Bees were chopped up with a serological pipette, and then the suspensions of bees and hive debris were bead-beaten in a TissueLyser II (Qiagen, Milan, Italy) at 30 Hz for 2 min. The homogenate was centrifuged at 14,000× *g* for 10 min at 4 °C, and the supernatant was transferred into an Eppendorf tube containing 1 mL of ice-cold isopropanol (Merck, Darmstadt, Germany), 25 µg of the RNA extraction control homogenized in RNase-free water and 5 µg of poly-A carrier RNA (Qiagen, Milan, Italy). The RNA extraction control was composed of *Tenebrio molitor* larvae obtained from a lyophilized food preparation intended for human consumption (Tarme della Farina Liofilizzate, ZIRP Insects, Vienna, Austria) and previously tested for the absence of amplification with honeybee-virus-targeted RT-qPCR assays. This insect sample was purchased in form of a commercial food preparation due to the unavailability of collections providing it. Aliquots of the RNA extraction control preparation were stored at −80 °C for future usage.

The Nucleozol/isopropanol suspension was agitated by inverting the tube and kept on ice for 30 min. Then, it was centrifuged at 14,000× *g* for 10 min, and the supernatant was discarded. The pellet was resuspended in 500 µL of Macherey Nagel MDB buffer (Carlo Erba, Cornaredo, Italy) and loaded into a New England Biolabs Monarch RNA Cleanup Column (Euroclone, Pero, Italy). The column was centrifuged for 1 min at 11,000× *g*, and the eluate was discarded. Then, the column was washed with 600 µL of Macherey Nagel RAW2 buffer (Carlo Erba, Cornaredo, Italy) and with 600 µL of ice cold 75% ethanol prepared with RNase-free water. The ethanol was completely removed by centrifugation at 11,000× *g* for 2 min, and the RNA was eluted in two steps with 30 µL of RNase-free water each for a total elution volume of 60 µL. The RNA was soon analyzed by RT-qPCR or stored at −80 °C until analysis.

The RNA extract obtained from the hive debris appeared dark for some samples; in this case, downstream amplification was inhibited. Therefore, these extracts were purified by adding 700 µL of 75% ethanol, incubating for 30 min in refrigerated conditions and centrifuging at 14,000× *g* for 15 min. Finally, ethanol was carefully and completely removed, and the pellet was resuspended in 60 µL of RNase-free water.

### 2.3. RT-qPCR

RT-qPCR was carried out in a QuantStudio 5 thermal cycler (Thermo Fisher Scientific) using the One-Step PrimeScript III RT-PCR Kit Takara Bio (Diatech, Jesi, Italy) master mix. The 20 µL reaction contained 10 µL of the master mix, 0.05 µg/µL BSA, 1× ROX, 0.2 µM of each primer and probe for the viral targets, and 0.1 µM of each primer and probe for the RNA extraction control derived from those described by Köppel et al. [49] (TeneF: 5′-CCATGAGTACGAATAAGAGAAACCAA-3′, TeneR: 5′-TTTAAGGCTTGAATTTGTTGTTTTATCTGTTTATT-3′ and the TeneP: 5′-JOE-AATAGATAGACCAAGAACGCCTTCACA-BHQ13′ probe), 4 µL of RNA extract and RNase-free water to adjust the reaction volume. A negative extraction control obtained without the sample and a positive control reaction containing 6 Log copies of synthetic RNA were included in each PCR run.

The PCR program was unique for all the targets and comprised 15 min at 50 °C for reverse transcription, 2 min of denaturation at 95 °C and 50 cycles of denaturation at 95 °C for 15 s and annealing at 54 °C for 30 s.

### 2.4. Determination of the Limit of Detection and Construction of Calibration Curves

The limit of detection (LOD), i.e., the lowest template concentration at which 95% of positive samples were correctly identified (LOD_95_), of the RT-qPCR tests was determined by using RNA fragments synthetized by GenScript Biotech (Rijswijk, The Netherlands) with sequences identical to the viral gene regions between the primers. The synthetic RNA fragments were quantified by the Qubit 3 Fluorometer (Thermo Fisher Scientific, Rodano, Italy) and the Qubit RNA HS Assay Kit (Thermo Fisher Scientific) according to the manufacturer’s instructions and were used to spike honeybee or hive debris samples, which, in preliminary assays, tested negative in the RT-qPCR tests to be evaluated in this study. The synthetic RNA controls were received in five aliquots and were stored at −20 °C, according to the manufacturer’s recommendations. These were used to prepare fresh serial dilutions in RNase-free water for sample spiking in each experiment. The quality of the synthetic RNA controls was checked at each use by the Qubit 3 fluorometer.

Twenty-four real samples for both honeybees and hive debris were collected from local beekeepers and analyzed to select those testing negative in the assays under study for use in the spiking experiments. These were collected in 100 mL sterile containers, transported in refrigerated conditions to the laboratory and stored at −80 °C until analysis. The copy number of the synthetic RNA was determined based on the concentration and length by using the online tool https://nebiocalculator.neb.com/#!/ssrnaamt (accessed on 25 January 2024). Serial dilutions of the synthetic RNA fragments were used to construct calibration curves by spiking honeybee or hive debris aliquots in triplicate. The LOD_95_ values for one bee and 100 µL of hive debris were expressed as the Log of the copy number and determined experimentally. The theoretical Log copy number per reaction was calculated as the (spiked copy number × 0.75/60) × 4 for bees and the (spiked copy number × 0.60/60) × 4 for hive debris, where 0.75 or 0.60 is the fraction of Nucleozol supernatant recovered after centrifugation of the honeybees or hive debris, respectively, and processed further in the RNA extraction; 60 is the elution volume in µL, and 4 is the number of µL used in the RT-qPCR reaction.

### 2.5. Statistical Analyses

The Student’s *t* test on Ct values was used to evaluate the effect of multiplexing on the amplification yield and sensitivity using Microsoft Excel 2016.

## 3. Results

### 3.1. In Silico Assessment of Existing Honeybee Virus Detection Methods

The outcomes of the in silico evaluation for the specificity and inclusivity of previously described methods suggested the design of new tests for ABPV and CBPV and the modification of existing methods for BQCV, DWVA, DWVB and SBV in order to include an higher number of virus variants. In particular, for BQCV, the primers and probe were derived from those described by Chantawannakul et al. [39] but shortened to fit the annealing temperature of the amplification program that was common to all the assays evaluated in this study. Reverse primers targeting DWVA and DWVB were the same as those described by Kevill et al. [38], while the forward primers and probes specific for each DWV variant were newly designed. The forward primer and the probe for SBV detection were derived from those validated by Blanchard et al. [41], while the reverse primer was newly designed. The primer/probes systems evaluated in this study are reported in Table 1.

The number of database entries perfectly matched by the primers reported in Table 1 were 91 for the ABPV-specific test, 496 for the BQCV-specific test, 110 for the CBPV-specific test, 256 for the DWVA-specific test, 129 for the DWVB-specific test and 232 for the SBV-specific test. These numbers correspond to those of the primers with the lowest number of database matches in each test. The inclusivity of the probes and primers with degenerate nucleotide positions was tested by BLAST runs with all the possible versions without degenerate positions. For all the specific tests, the theoretical number of matched sequences was higher than for the previously reported assays. The DWVA-specific test, like those reported previously, also matches with the Kakugo variant [50].

### 3.2. Multiplexing of RT-qPCR Tests Targeting Honeybee Viruses

The RT-qPCR assays designed in this study were tested for their multiplexing potential in order to reduce the number of analyses necessary to diagnose the six viral pathogens considered. Therefore, duplex reactions were tested on two synthetic target RNAs combined at the same concentration or with one in great excess compared with the other.

The RNA extraction control used in the reactions was constituted of a homogenate of *T. molitor*. The decision to use this organism was based on the possibility of extending its future use also to honeybee insect parasites and on the low probability of its presence in hives. Moreover, a qPCR-specific test was available for its detection [49]. Duplex RT-qPCR reactions carried out without the synthetic RNA targets demonstrated the absence of false-positive results deriving from the RNA extraction control.

The best-performing RT-qPCR assay combinations were ABPV with BQCV, DWVA with DWVB and CBPV with SBV. The Student’s t test indicated that when the templates were present at the same concentration, the difference between the series of Ct values obtained in triplicate reactions containing only one or both targets in most cases was not statistically significant over the range 1–6 Log copy numbers. The *p* values obtained for the Ct comparison of each target alone or in association with the other target at the different concentrations are reported in Table 2.

As shown, statistically different Ct series for *p* < 0.05 were obtained only for the DWVA-targeted test at the lowest concentration. This was caused by the increase in Ct values in the duplex reaction. However, in the duplex reaction, the target virus was also detected in all replicates at the lowest concentration, so the sensitivity did not vary compared to the reaction with the DWVA target alone. The experiments comparing the amplification yield between reactions containing one or two targets were also carried out using both the primer/probe systems for the duplex test when only one target was present. This highlighted that the DWVA- and DWVB-targeted tests were not specific for the intended variant. Indeed, the DWVA-targeted test gave rise to an apparent DWVB-specific amplification at Ct values 8 ± 1 lower than those of the DWVA target when only the latter was present. Moreover, the DWVB-specific test gave rise to an apparent DWVA-specific amplification at Ct values 4 ± 1 lower than those of the DWVB target. This could be a consequence of the lack of specificity of the probes or primers when the amplification product accumulates, despite this not being evident in the in silico assessment of specificity. Duplex RT-qPCR runs carried out with one of the two targets at a higher concentration allowed us to determine that DWVB cannot be specifically detected in real samples when present at more than 2 Log copy numbers lower than DWVA, and DWVA cannot be determined in real samples by the duplex reaction when present at more than 1 Log copy number lower than DWVA. Nonetheless, the duplex test identifies the DWV variant that predominates in a sample and can detect coinfection with DWVA and DWVB when the latter is not lower than 2 Log copy numbers compared with DWVA and when DWVA is not lower than 1 Log copy number compared with DWVB. The detection of both variants was not affected when these were present at the same concentration, thus allowing the construction of calibration curves. For the reliable detection and quantification of the two DWV variants in real samples, the use of separate singleplex reactions is advisable. Alternatively, different combinations in the duplex reactions were tested, and the associations in the BQCV/DWVA and ABPV/DWVB assays showed no significant differences in amplification yield with different template concentrations compared with the singleplex reactions.

It was also observed that the BQCV-specific test sometimes presented abnormal amplification curves for template amounts higher than 6 Log copy numbers. Since the amplification curve initially shows a normal trend, when using the BQCV-specific test, it is opportune to register the Ct values during the run in the analysis of real samples to identify those with high viral loads.

### 3.3. Limit of Detection (LOD) of the RT-qPCR Tests

The LOD of the RT-qPCR tests designed in this study was determined both in terms of the template Log copy number detectable in the PCR reaction and in terms of the Log copy number spiked in honeybee and hive debris samples. According to the minimum requirement guidelines (MIQE) for the publication of qPCR tests [51], the LOD_95_ was defined. Therefore, 20 replicate reactions using synthetic RNA targets or 20 extractions from spiked bees and hive debris were carried out, followed by the RT-qPCR tests to define the LOD. To this aim, five extraction/amplification replicates were preliminarily tested at decreasing target concentrations, and the lowest concentration allowing amplification from all five extracts was finally used in extractions/amplifications from 20 replicates. LOD_95_ values obtained for each viral target in the duplex reactions are shown in Table 3.

The LOD_95_ was also confirmed in duplex reactions with different concentrations of the two templates to define if competition between targets could decrease the sensitivity. In these duplex reactions, one template was present at 5 Log copy numbers more than the target at the LOD_95_ concentration, and no loss in sensitivity was observed for the latter. Moreover, the LOD_95_ was unvaried for ABPV, BQCV, DWVA and DWVB in the BQCV/DWVA and ABPV/DWVB assays.

### 3.4. Virus Quantification in Artificially Spiked Samples

**Figure 1:**
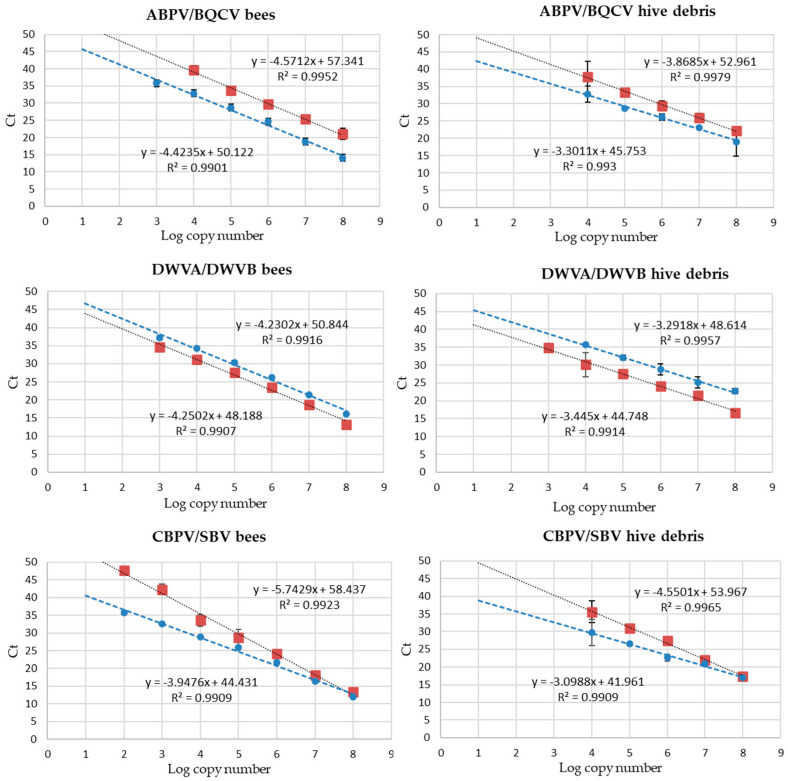
Standard curves for the quantification of viruses in honeybees and hive debris in duplex RT-qPCR reactions. Red symbols: ABPV, CBPV, DWVA; blue symbols: BQCV, DWVB, SBV. Ct values are shown for the linearity range of the calibration curves.

Calibration curves for the quantification of viral loads by the duplex RT-qPCR reactions targeted at ABPV/BQCV, DWVA/DWVB and CBPV/SBV were constructed for the honeybee and hive debris samples and presented a linearity range encompassing at least five decimal dilutions of the target synthetic RNA. Examples are shown in Figure 1.

Results showed that the RT-qPCR tests evaluated in this study can be applied to quantify viral loads ranging between 2/4 and 8 Log copy numbers in bees and hive debris. In the BQCV/DWVA and ABPV/DWVB assays, ABPV-, BQCV-, DWVA- and DWVB-targeted tests showed the same linearity ranges and similar efficiencies to those reported in Figure 1, with slope values of 4.3156, 4.4562, 4.2134 and 4.1581, respectively, for honeybees and 3.4562, 3.2567, 3.2789 and 3.3345, respectively, for hive debris, with an R^2^ above 0.99.

The first application to real samples regarded the honeybees and hive debris used to select those negative to the tests under evaluation to be used for spiking. Of the 24 honeybee samples analyzed, 3 tested negative for all viruses and 1 was positive for only DWVA. All other samples showed co-infection with two (eight samples), three (five samples) or four (seven samples) viruses. Among the 24 hive debris samples, 2 were negative for all viruses, 2 were positive for one virus (DWVA or BQCV), 1 for two viruses, 11 for three viruses and 8 for five viruses. ABPV was detected in six hive debris samples at levels close to LOD_95_ but in none of the honeybee samples. BQCV and CBPV were the viruses most frequently detected. When quantified by reference to the calibration curves from honeybees and hive debris (Figure 1), the viral loads in most samples ranged between below LOD_95_ to about 6 Log copy numbers, and two honeybee samples showed viral loads exceeding 7 Log copy numbers for DWVA and DWVB, respectively.

## 4. Discussion

This study, undertaken to select the most specific and inclusive methods among those described for the detection of the most relevant honeybee viruses by RT-qPCR, led to the modification of some existing methods or the design of new ones to extend the possibility of detecting a higher number of virus variants. The finding that some previously reported tests were not sufficiently specific or partially inclusive is explained by the low number of target sequences available at the time when the assays were designed. This observation indicates the necessity of periodically re-evaluating molecular detection methods against recently acquired sequence data.

In this study, duplex RT-qPCR reactions in the presence of an RNA extraction control for honeybee viruses were performed for the first time and gave good results in terms of sensitivity and linearity range for all the targets. Therefore, the possibility of carrying out different determinations in a single assay was demonstrated, with the advantages of rapidity and a reduction in the amount of work and costs.

The cross-reactivity of the DWVA-specific test toward DWVB, and vice versa, can be explained by the high nucleotide identity (89%) between these two variants [52]. This problem was unforeseen based on the BLASTN alignment of primers and probes with the sequence of the non-target variants, so experimental verification was fundamental to evaluating the specificity of the qPCR assay. Using a different target region in the DWV viral genome would have not necessarily avoided cross-reactivity because of the high sequence similarity between the two viral variants.

On the other hand, the cross-reactivity of the test described here does not compromise the detection of the dominant DWV variant in a sample. Since DWVB cannot be detected in the duplex reactions when its copy number is more than 2 Logs lower than that of DWVA, and DWVA cannot be detected in the duplex reactions when its copy number is more than 1 Log lower than DWVB, the use of singleplex reactions can allow the detection of the less abundant variant with certainty. Alternatively, different combinations of tests in the duplex reactions, namely BQCV/DWVA and ABPV/DWVB, can be used.

The sensitivity reached in the virus-specific tests in this study can be sufficient to detect honeybee viral infections in real samples since, for instance, the predicted mean viral load for DWVB is 1.5 × 10^4^ viral particles per bee on *Varroa*-free sites for *A. mellifera* [21]. As observed in the amplifications from real samples, even lower viral levels than this can be detected.

Most tests, particularly the CBPV-specific assay, showed a low efficiency when applied to honeybees, although a wide linearity range was observed and the LOD_95_ was comparable among the assays. PCR efficiency is dependent on a number of factors, including operating conditions and aspects of the target region and primer/probe sets such as secondary structures in the vicinity or within the target region, the efficiency of priming and the tendency of primers to dimerize. Moreover, efficiency is known to vary for each test and each matrix [51,53,54]. In this case, the presence of interfering abundant nucleic acids from honeybees and their microbiome could be the cause of the PCR efficiency drop in all tests when applied to these samples (Figure 1).

However, acceptable sensitivity was obtained despite the low efficiency of the RT-qPCR reactions, and positive results were observed after cycle 40 for most assays. Use of 50 cycles in a PCR program in probe-based detection is not expected to give rise to false positives due to PCR artifacts. Moreover, this possibility was excluded experimentally by performing a negative control reaction in each run. This number of cycles was used also in other studies [55].

The RT-qPCR methods described in this study also showed a sufficiently wide linearity range in virus detection from hive debris, thus proving useful for future non-invasive diagnoses of honeybee viral infections. Indeed, the collection of hive debris from the hive bottom drawer is a simple method for beekeepers to obtain samples suitable for the diagnosis of microbial diseases without the need to sacrifice honeybees for the analysis. Therefore, the use of this matrix could lead to more frequent testing and better monitoring of apiary health, even for preventive purposes.

Considering the good inclusivity of the modified or newly designed tests, it is possible to foresee their application in prevalence investigations and in the identification of infection-associated factors, with the aim of protecting honeybees or other susceptible Apidae pollinators from these pathogens. The definition of the overall viral infection status, also in relation to the presence of other hive diseases and to the strength of the honeybee colony, would shed light on the detrimental effects caused by these pathogens and on how to reduce their occurrence.

The extraction methods and the RT-qPCR tests described could be adapted to other difficult solid hive matrices such as honey and pollen, since these materials have been shown to better reflect the viral profile of forager bees [56].

The assays described here can further be improved by their application together with a recently developed semi-automated magnetic-beads-based technology applied to DWVA and DWVB RNA extraction from honeybee samples that allows for the rapid screening of viral loads in 96 samples simultaneously [47].

## Figures and Tables

**Table 1:** Sequences of primers and probes experimentally tested in this study for each honeybee virus, showing their target genes and annealing positions on whole-genome database entries.

Label	Sequence 5′-3′ *	Target	Nucleotide Positions	Reference
	Acute paralysis virus (ABPV)			
APVF	TTTGTTTCAAAYAARATGTTYATGAAAYC	Capsid protein gene	Acc. no. ON648748.1 8324-8466	This study
APVP	FAM-TATGGTGGAAAYKCTGARAAYAAT-MGBEQ			
APVR	BTWGAHACAGTCTCTGGACACAT			
	Black queen cell virus (BQCV)			
BQCVF	GTGCGGGAGATGATATGGA	Capsid protein gene	Acc. no. MT482476.1 8060-8128	This study [39]
BQCVP	Cy5-TTTCCATCTTTATCGGTACGC-MGBEQ			
BQCVR	CCGTCTGAGATGCATGAATAC			
	Chronic bee paralysis virus (CBPV)			
CBPVF	GAAGTCATCCGTAGATCTGG	RNA1 gene	Acc. no. MK637522.1 1961-2070	This study
CBPVP	FAM-AGACKAGRGAGGAYGGGA-MGBEQ			
CBPVR	CRAGAGGGGTATGTTGTACT			
	Deformed wing virus A (DWVA)			
DWVAF	CTTTGTCTTCATTAAAGCCAC	Polyprotein gene	Acc. no. OR497397.1 8636-8774	This study [38]
DWVAP	FAM-TGCGTGGAATGCGTCC-MGBEQ			
DWVAR	CTCATTAACTGTGTCGTTGAT			
	Deformed wing virus B (DWVB)			
DWVBF	TTTATCTTCATTAAAACCGCCA	Polyprotein gene	Acc. no. OR497394.1 8615-8752	This study [38]
DWVBP	Cy5-ATCTTTTGAGAGGGATGAGA-MGBEQ			
DWVBR	CTCATTAACTGAGTTGTTGTC			
	Sacbrood virus (SBV)			
SBVF	AAYGTCCACTACACCGAAATGT	Polyprotein gene	Acc. no. MN082652.1 430-548	This study [41]
SBVP	Cy5-TGATGAGAGTGGACGAAGAATCTGGAATG-BHQ2			
SBVR	TAHGAGGTAATAACTTTTCGCCA			

* degenerate nucleotide position code: B (C,G,T); H (A,C,T); K (G,T); R (A,G); W (A,T); Y (C,T); (https://www.bioinformatics.org/sms/iupac.html, accessed on 24 April 2024).

**Table 2:** *p* values obtained by the Student’s t test in the comparison of Ct values obtained in triplicate reactions containing one or both the synthetic target RNAs.

	Log Copy Number					
	1	2	3	4	5	6
Target	*p* Values					
ABPV	0.91	0.90	0.98	0.92	0.31	0.33
BQCV	0.90	0.87	0.51	0.96	0.53	0.10
CBPV	0.98	0.05	0.22	0.72	0.15	0.58
DWVA	0.02 *	0.44	0.97	0.78	0.08	0.37
DWVB	0.16	0.18	0.48	0.75	0.15	0.57
SBV	0.51	0.80	0.79	0.28	0.91	0.65

* statistically significant for *p* < 0.05.

**Table 3:** LOD_95_ values for each viral target in the duplex RT-qPCR tests carried out on solutions of the synthetic RNA targets and on RNA extracts from honeybees and hive debris samples spiked with the synthetic RNA targets.

	Log Copy Number		
	PCR Reaction	One Bee	100 µL Hive Debris *
Target			
ABPV	1.99	3.65	3.47
BQCV	1.37	2.81	2.88
CBPV	1.21	2.9	2.72
DWVA	1.25	2.95	2.77
DWVB	1.39	4.07	3.90
SBV	1.49	3.17	4.00

* approximate volume of hive debris.

## Data Availability

The original contributions presented in the study are included in the article, further inquiries can be directed to the corresponding author.
